# Augmentation of psychrophilic anaerobic digestion with psychrotolerant *Serratia marcescens*, calcium phosphate (CaHPO_4_·2H_2_O) and hematite (α-Fe_2_O_3_) nano-additives

**DOI:** 10.3389/fmicb.2026.1756298

**Published:** 2026-02-25

**Authors:** Haripriya Rama, Busiswa Ndaba, Mokhotjwa Simon Dhlamini, Malik Maaza, Nicolene Cochrane, Ashira Roopnarain

**Affiliations:** 1 Microbiology and Environmental Biotechnology Research Group, Agricultural Research Council – Natural Resources and Engineering, Pretoria, South Africa; 2 Department of Physics, College of Science, Engineering and Technology, University of South Africa – Florida Campus, Johannesburg, South Africa; 3 Institute for Catalysis and Energy Solutions, College of Science, Engineering and Technology, University of South Africa – Florida Campus, Johannesburg, South Africa; 4 iThemba LABS, Nanosciences African Network Materials Research Department, National Research Foundation of South Africa, Cape Town, South Africa; 5 UNESCO-UNISA iTLABS/NRF Africa Chair in Nanosciences-Nanotechnology, College of Graduate Studies, University of South Africa, Pretoria, South Africa; 6 AGRIMETRICS, Agricultural Research Council – Biometry, Pretoria, South Africa; 7 Department of Environmental Sciences, College of Agriculture and Environmental Sciences, University of South Africa – Florida Campus, Johannesburg, South Africa

**Keywords:** anaerobic digestion, bioaugmentation, biostimulation, low temperature, microbial communities, *Sclerocarya birrea*, *Serratia marcescens*

## Abstract

Psychrophilic anaerobic digestion (PAD) requires optimization to improve methane production at low temperatures (<20 °C). This study aimed to improve methane production via bioaugmentation with psychrotolerant *Serratia marcescens* (SM) and biostimulation with nano-additives, comprising calcium phosphate (CaP) and hematite (α-Fe_2_O_3_) nanoparticles (NPs), during batch PAD of cattle manure and food waste at 15 °C. The highest methane yields were obtained from treatment with SM and both NPs (163.9 ± 18.0 mL CH_4_ g^−1^ VS), thereafter with the combination of CaP and α-Fe_2_O_3_ NPs (143.9 ± 50.2 mL CH_4_ g^−1^ VS). The lowest yield was observed in the control (70.2 ± 4.9 mL CH_4_ g^−1^ VS) followed by treatment with SM alone (124.6 ± 20.3 mL CH_4_ g^−1^ VS). Treatment with CaP and α-Fe_2_O_3_ NPs reduced the lag phase more than the other treatments. Moreover, the addition of nano-additives biostimulated PAD without significantly altering the microbial community composition. The dominant genera included *Bacteroides, Acinetobacter*, and *Methanosarcina* (a mixotrophic methanogen) after batch PAD across all treatments. This research provides new insights on the augmentative effect of SM, CaP and α-Fe_2_O_3_ NPs on methane production and microbial community dynamics during PAD.

## 1 Introduction

Anaerobic digestion (AD) has been identified as a circular approach for renewable energy generation through organic waste valorisation. During this microbial-driven process, nutrients and biogas (containing combustible methane) can be recovered from organic waste such as animal manure, municipal sewage and food waste (Emmanuel et al., 2024). Methane production during the AD process primarily depends on interspecies electron transfer (IET) via hydrogen or formate transfer (IHT/IFT), which act as electron carriers between syntrophic fermentative bacteria and methanogenic archaea. Elevated hydrogen partial pressure may inhibit syntrophic metabolism and methanogenesis by disrupting the delicate thermodynamic balance required for IHT (Xu et al., 2019; Feng et al., 2023). Only when hydrogen partial pressure is low enough whereby hydrogen is consumed by hydrogenotrophic methanogens, syntrophic metabolism and methanogenesis becomes thermodynamically favorable (Feng et al., 2023). Furthermore, slower transport of electrons between syntrophic bacteria and methanogens may occur when low concentrations of electron carriers (hydrogen or formate) are available, thus IET can become the bottleneck during methanogenesis (Kato et al., 2012). In addition, environmental factors such as temperature and pH can affect methane production via IHT/IFT (Hoffmann et al., 2022). These factors can lead to altered composition and metabolic efficiencies of the AD microbial community (Xu et al., 2023).

Conductive material as well as certain pili and c-type cytochrome outer membrane proteins can accelerate extracellular electron transfer (EET) from syntrophic bacteria, during oxidation of organic substrates, to methanogens via direct interspecies electron transfer (DIET; Zhu et al., 2022). Kato et al. (2012) were the first to report on the ability of (semi)conductive magnetite (Fe_3_O_4_) and α-Fe_2_O_3_ iron oxide nanoparticles, synthesized using chemical co-precipitation methods, to facilitate methanogenesis via syntrophic DIET interactions between electroactive bacteria and electrotrophic methanogens (Kato et al., 2012). In their study, syntrophic *Geobacter*, which are generally dissimilatory iron-reducing bacteria possessing outer membrane c-type cytochromes, were shown to interact with Fe_3_O_4_ and α-Fe_2_O_3_ nanoparticles by utilizing them as conduits for electron transfer to *Methanosarcina* methanogens, thus accelerating methanogenesis (Kato et al., 2012). Since then, several review articles have reported on the conductive nature of iron oxide nanoparticles, which can promote DIET between syntrophic bacteria and methanogens for enhanced methane production during mesophilic and thermophilic AD (Gahlot et al., 2020; Pasalari et al., 2021; Chen et al., 2022; Wu et al., 2023).

Additionally, various trace element metallic nanoparticles were reported to have stimulating effects on the AD microbiome with respect to metabolic activities, growth rates and cell division subsequently resulting in enhanced methane production during mesophilic AD (Abdelsalam and Samer, 2019; Juntupally et al., 2023). Metallic nanoparticles can promote methane production at lower concentrations compared to their larger-sized counterparts (Jadhav et al., 2022). Iron and calcium ions, which can be derived from trace elemental nanoparticles, are necessary for improving methane production during AD (Casals et al., 2014). The growth and activity of microorganisms are dependent on elements such as iron, which form part of essential co-factors and enzymes that stabilize AD performance, in terms of methane production, by stimulating acidogenesis and methanogenesis (Abdelsalam and Samer, 2019). On the other hand, calcium plays an important role in bacterial cell regulation, while inorganic phosphorus sources such as calcium phosphate can promote microbial diversity and growth due to its efficient utilization by most microorganisms (Dominguez, 2004; Zheng et al., 2019).

Typically, mesophilic (20–45 °C) and thermophilic AD (45–60 °C) have higher methane production efficiencies than AD conducted under low temperatures (<20 °C) (Rodríguez-Jiménez et al., 2022). However, much of the methane generated from mesophilic and thermophilic AD is expended to maintain those high temperatures, particularly in colder regions (Xu et al., 2023). Furthermore, most household scale biogas technologies adopted across the world utilize low-cost digesters, such as the fixed-dome model, which operate at ambient temperatures and are void of expensive, active heating systems (Martí-Herrero et al., 2022). Although psychrophilic AD (PAD) provides environmental benefits and demands less energy/operating costs for temperature maintenance, lower temperatures can affect the composition and metabolic efficiencies of the AD microbial community (Xu et al., 2023).

Several reports have highlighted the research need to investigate and optimize psychrophilic AD (PAD), particularly using nanotechnology, bioaugmentation, cold-adaptation and co-digestion approaches (Dev et al., 2019; Yao et al., 2020; Tiwari et al., 2021; Akindolire et al., 2022; Rodríguez-Jiménez et al., 2022). The PAD fermentation cycle is generally much longer due to the inhibiting effects of low temperatures on AD microbiome activity (Martí-Herrero et al., 2022). Bioaugmentation with selected psychrophilic strains/consortia can potentially reduce start-up time and ensure stable daily methane production via PAD (Tiwari et al., 2021; Akindolire et al., 2022). Obi et al. sequenced the whole genome of *Serratia marcescens* strain 39_11H, which was shown to enhance mesophilic AD of cattle manure and water hyacinth due to its hydrolytic and acidogenic properties (Obi et al., 2020). The whole genome sequence data of the isolate revealed the presence of genes related to hydrolysis and acidogenesis as well as genes for cold tolerance.

While several studies have explored the impact of bioaugmentation of PAD using microbial consortia (Xu et al., 2022, 2023; Yan et al., 2023; Shang et al., 2025), few have been conducted on the use of a single bacterial isolate, such as *S. marcescens*, to bioaugment PAD (Akindolire et al., 2022). Hence, this study investigated the effect of SM on batch PAD of cattle manure (CM) and food waste (FW) at 15 °C. Additionally, this study investigated the application of SM alongside α-Fe_2_O_3_ and/or CaP NPs for enhancing PAD. Metallic nano-additives can be synthesized using physical, chemical and biological methods, however there are many benefits of utilizing the biological approach for synthesis (Husen and Siddiqi, 2014; Hassan et al., 2018; Rabalao et al., 2022). For instance, plant-mediated synthesis can promote nanoparticle production by utilizing the natural reducing and capping agents present in the extracts of plant-based waste materials to reduce metal ions arising from pre-cursor metal salts to nanoparticles (Ndaba et al., 2022), while further supporting the notion of a circular economy. In our previous work, extracts of *Sclerocarya birrea* shell waste were shown to be an effective source of reducing and capping agents to form α-Fe_2_O_3_ and CaP NPs from pre-cursor metal salts (Rama et al., 2023, 2025). Iron and calcium are considered redox-active metals that can participate in IET between syntrophic bacteria and methanogens to enhance methane production (Yang et al., 2021), hence *S. birrea*-mediated α-Fe_2_O_3_ and CaP NPs were selected for this investigation. Furthermore, studies investigating the combined effect of conductive and biostimulating nanoparticles, such as α-Fe_2_O_3_ and CaP, on methane production and microbial community composition during PAD are rare. Therefore, it was hypothesized that the combined effects of the additives on methane production would be greater than individual effects. The present study is the first known study to determine the individual and dual effects of SM, CaP NPs and α-Fe_2_O_3_ NPs on PAD. The findings of this study provide further insight on the potential applicability of the additives, and their effects on the microbial communities, in augmented PAD systems.

## 2 Materials and methods

### 2.1 Preparation of calcium phosphate and hematite nano-additives

The *S. birrea*-mediated nanoparticles were produced in our previous work (Rama et al., 2025). Briefly, *S. birrea* shell powder was boiled for 30 min in deionized water (dH_2_O; 25 g L^−1^) with continuous stirring. The liquid extract was separated from the shell powder by centrifugation (10 min, 2,683 × *g*) using a Digital Centrifuge DSC-301SD (New Taipei City, Taiwan) and filtered using Whatman 52 filter paper (Rama et al., 2023). The *S. birrea*-mediated CaP NPs were prepared previously (Rama et al., 2025), whereby calcium nitrate (Ca(NO_3_)_2_· × H_2_O, Merck, Darmstadt, Germany) and anhydrous disodium hydrogen orthophosphate (Na_2_HPO_4_, Merck, Darmstadt, Germany) salts were added to the extract (0.1 mol L^−1^) and stirred for 30 min. The CaP NPs were washed thrice with dH_2_O and centrifuged (10 min, 2,683 × *g*). The washed CaP NPs were oven-dried overnight at 100 °C. The *S. birrea*-mediated α-Fe_2_O_3_ NPs were also prepared previously (Rama et al., 2025), whereby iron nitrate (Fe(NO_3_)_3_·9H_2_O, Merck, Darmstadt, Germany) precursor salt was added to *S. birrea* shell extract (0.1 mol L^−1^). The mixture was adjusted to pH 10–11 and was stirred continuously for 30 min. The α-Fe_2_O_3_ NPs were washed thrice with dH_2_O and centrifuged (10 min, 2,683 × *g*). Thereafter, α-Fe_2_O_3_ NPs were oven-dried overnight at 100 °C and annealed at 500 °C for 2 h. The characterization of the NPs was conducted previously (Rama et al., 2025). The characteristics of the NPs used in this study is summarized in Supplementary Table 1. Based on the elemental composition determined using energy-dispersive X-ray analysis, pure CaP and α-Fe_2_O_3_ NPs had formed and were capped with a negligible amount of carbon originating from the *S. birrea* extract (Rama et al., 2025).

### 2.2 Preparation of psychrotolerant *Serratia marcescens*

A glycerol stock (1 mL) of *S. marcescens* strain 39_H1 obtained from a previous AD study (Obi et al., 2020), was added to 39 mL of nutrient broth (NB; Merck, Darmstadt, Germany) in a 100 mL borosilicate bottle, aseptically, and was cultured at 150 rpm under aerobic conditions using a low-temperature shaking incubator (IncoShake, Labotec, Johannesburg, South Africa). This was conducted initially at 30 °C (24 h), thereafter at 25 °C (24 h), 20 °C (48 h) and 15 °C (72 h) by subculturing 1 mL of each preceding culture into 49 mL of sterile NB. The resulting psychrotolerant *S. marcescens* (SM) that was cultured at 15 °C was further sub-cultured on sterile nutrient agar (NA) plates (Merck, Darmstadt, Germany) for 48 h to obtain isolated colonies of SM (Supplementary Figure 1). The concentration of colony forming units per milliliter (CFU mL^−1^) of SM culture was determined using a standard concentration curve prepared as previously described (Campbell, 2010).

### 2.3 Substrates and inoculum

The FW, CM and cold-adapted inoculum were obtained and characterized in our previous work (Rama et al., 2024). Briefly, market-rejected FW (peppers, baby onions, beetroots, cucumbers, carrots, cabbage, broccoli and lettuce) obtained from Tshwane Market (Pretoria, South Africa) was homogenized using a blender, pasteurized at 70 °C for 1 h and stored at −20 °C until further use. Fresh cattle manure that was aseptically collected from the Agricultural Research Council—Animal Production (Pretoria, South Africa) was mixed and stored at −20 °C until further use. The cold-adapted inoculum was prepared by mixing 33.3 g CM and 16.7 g FW in a working volume of 500 mL in a high pressure resistant, airtight 1,180 mL borosilicate glass reactor closed with a polytetrafluoroethylene coated septum. The reactor was successively incubated at 30 °C (1 week), 25 °C (1 week), 20 °C (2 weeks) and 15 °C (10 weeks), with shaking at 100 rpm (Low temperature IncoShake Incubator, Labotec). Thereafter, the cold-adapted inoculum was stored at 4 °C prior to use on the same day for the batch culture trials. The inoculum was primarily composed of *Methanosarcina, Methanobrevibacter, Methanobacterium, Macellibacteroides, Bifidobacterium, Clostridium sensu stricto, Bacteroides* and *Acinetobacter* (Rama et al., 2024).

### 2.4 Psychrophilic anaerobic digestion batch culture trials

Batch cultures were prepared in triplicate in airtight 366 mL borosilicate bottles with PTFE coated septa and a working volume of 150 mL. The control batch cultures contained 10% (w/v) CM, FW and cold-adapted inoculum (2:1:1). The experimental batch cultures contained the same components as the control, however with the addition of SM, 50 mg L^−1^ CaP NPs and/or 25 mg L^−1^ α-Fe_2_O_3_ NPs, in different combinations. The compositions of the batch cultures are shown in Table 1. The batch cultures were conducted using an inoculum to substrate ratio (ISR) of 0.09 g VS inoculum g^−1^ VS substrate. While this ratio is lower than recommended ratios for standardized tests (Holliger et al., 2016), stable and uninhibited digestion was observed throughout the experimental period. The concentrations of the NPs were selected based on the findings of previous research (Li et al., 2021; Akar et al., 2025), and were added to the batch cultures in powder form. Additionally, SM treated batch cultures contained 6% (v/v) SM (5 × 10^9^ CFU mL^−1^) based on concentrations applied in a previous study (Zhang et al., 2018). Briefly, SM cultured at 15 °C in NB, as per Section 2.2, was diluted with sterile NB until the optical density at 600 nm corresponded to 5 × 10^9^ CFU mL^−1^ using the standard concentration curve (Supplementary Figure 2). The final volume of the diluted culture was recorded as *V*_*f*_. Then, the diluted culture was pelleted and rinsed thrice in sterile dH_2_O (10 min, 2,683 × *g*). The cells were resuspended in sterile dH_2_O, whereby the final volume was the same as *V*_*f*_, and was used immediately for preparation of SM treated batch cultures. The final volume of SM in SM treated batch cultures was 4 mL. The batch cultures were run for 103 days at 15 °C with 100 rpm shaking speed in a low-temperature shaking incubator (IncoShake, Labotec, Johannesburg, South Africa).

**Table 1:** Treatment compositions of the psychrophilic anaerobic digestion batch cultures.

**Parameter**	**C**	**CSM**	**CSC**	**CSF**	**CSCF**	**CC**	**CF**	**CCF**
pH	7.07	7.09	7.12	7.10	7.09	7.14	7.15	7.15
Working volume (mL)	150	150	150	150	150	150	150	150
Cattle manure (g)	7.50	7.50	7.50	7.50	7.50	7.50	7.50	7.50
Cattle manure (g TS)	1.21	1.21	1.21	1.21	1.21	1.21	1.21	1.21
Cattle manure (g VS)	0.98	0.98	0.98	0.98	0.98	0.98	0.98	0.98
Food waste (g)	3.75	3.75	3.75	3.75	3.75	3.75	3.75	3.75
Food waste (g TS)	0.25	0.25	0.25	0.25	0.25	0.25	0.25	0.25
Food waste (g VS)	0.20	0.20	0.20	0.20	0.20	0.20	0.20	0.20
Inoculum (g)	3.75	3.75	3.75	3.75	3.75	3.75	3.75	3.75
Inoculum (g TS)	0.12	0.12	0.12	0.12	0.12	0.12	0.12	0.12
Inoculum (g VS)	0.11	0.11	0.11	0.11	0.11	0.11	0.11	0.11
SM (% (v^*^/v))	0	6	6	6	6	0	0	0
CaP NPs (mg L^−1^)	0	0	50	0	50	50	0	50
α-Fe_2_O_3_ NPs (mg L^−1^)	0	0	0	25	25	0	25	25
TS%	1.67	1.23	1.15	1.33	1.26	1.38	1.50	1.24
VS (of TS; %)	89.47	95.24	83.33	90.48	78.26	87.50	76.92	80.95
ISR	0.09	0.09	0.09	0.09	0.09	0.09	0.09	0.09

* Volume contained 5 × 10^9^ CFU mL^−1^.

TS, total solids; VS, volatile solids; SM, psychrotolerant *Serratia marcescens*; CaP NPs, calcium phosphate nano-additives; α-Fe_2_O_3_ NPs, hematite nano-additives; ISR, inoculum to substrate ratio.

### 2.5 Analytical methods

The total solids (TS), volatile solids (VS), methane yield, pH, FOS/TAC (volatile fatty acids (VFAs) to total alkalinity) ratio, C/N (carbon/nitrogen) ratio as well as lipid, protein and carbohydrate content were determined according to methods described in our previous work (Rama et al., 2024). Headspace gas samples (2 mL) were collected from the high pressure-resistant batch bottles using gas-tight syringes and were analyzed for methane content by gas chromatography (GC) using an 8610C Gas Chromatograph (SRI Instruments, Torrance, CA, USA) equipped with a thermal conductivity detector and a HayeSep D packed column. Initially, gas samples were analyzed by GC once a week. Thereafter, samples were analyzed three times a week when methane was detected in the gas samples. The methane volume was calculated by multiplying the methane percentage obtained from the GC analysis by the headspace volume, and the methane yield was determined relative to the VS added as previously described (Nkuna et al., 2019). Methane volumes were estimated from the measured methane fraction and the known headspace volume under ambient pressure (1 atm) and standard temperature (15 °C). The pH, C/N, TS and VS (of TS) were 4.25, 17.11, 6.62% and 79.83% for FW; 8.11, 19.66, 16.11% and 80.99% for CM; and 7.05, 20.90, 3.19% and 90.70% for cold-adapted inoculum, respectively. The lipid, protein and carbohydrate content of CM and FW were 2.44%, 11.22% and 11.86%, and 1.08%, 13.95% and 7.71%, respectively (Rama et al., 2024). Analysis of variance (ANOVA) was conducted for the methane yields obtained using XLSTAT (standard) in Excel (Microsoft Inc., Paris, France), SAS9.4 for Windows (SAS Institute Inc., Cary, North Carolina, United States; SAS Institute, Inc., 2016). In addition, ANOVA with Tukey's post hoc test was conducted for the lag phases using R version 4.4.1 (R Core Team, 2021) in RStudio (RStudio Team, 2020).

### 2.6 Kinetics modeling

The methane production potentials for all the treated batch cultures were evaluated by applying the following modified Gompertz model (Equation 1) to fit the experimental methane yields:


$$\begin{array}{l} Y=P\times exp\left\{-exp\left[\frac{Rm\;\times \;2.718282}{P}\left(\lambda -t\right)+1\right]\right\} \end{array}$$

where *Y* is the methane yields (mL CH_4_ g^−1^ VS), t is the trial duration in days, P is the maximum methane production potential (mL CH_4_ g^−1^ VS), R is the maximum methane production rate (mL CH_4_ g^−1^ VS day^−1^) and λ is the lag phase period in days (Rama et al., 2024).

### 2.7 Microbial community analysis

Triplicate DNA extraction of the batch culture samples from before and after the trials was conducted using the ZymoBIOMICS DNA Miniprep Kit, according to manufacturer instructions (Zymo Research, Irvine, United States). Thereafter, 16S rRNA V4-V5 region amplification, amplicon sequencing and analyses were conducted as previously described (Rama et al., 2024). Briefly, DNA extracts were normalized to 2 ng μL^−1^ and the replicate extracts were pooled. Then, PCR amplification of the 16S rRNA V4-V5 gene region using the 515F-Y and 915R primers (Fadeev et al., 2021), containing Illumina adapters, and OneTaq^®^ 2× Master Mix with Standard Buffer (New England Biolabs Inc., Ipswich, MA, USA) was conducted with the T100 Thermal Cycler (Bio-Rad Laboratories, Hercules, CA, USA). The PCR conditions were as follows: 94 °C for 30 s (initial denaturation), 94 °C for 30 s (denaturation); 55 °C for 30 s (annealing) and 68 °C for 1 min (extension) for 35 cycles; and 68 °C for 5 min (final extension). The PCR products were sent to the Agricultural Research Council – Biotechnology Platform (Onderstepoort, South Africa), where they were cleaned, indexed and sequenced using the Illumina MiSeq benchtop system (San Diego, CA, USA). The raw sequences were trimmed and assessed for quality using Trimmomatic (Bolger et al., 2014) and FastQC (Andrews, 2010), respectively. Thereafter, feature tables of amplicon sequence variants, taxonomic classification, and alpha and beta diversities of the samples were obtained using the QIIME2™ (Bolyen et al., 2019) and DADA2 (Callahan et al., 2016) pipelines, and the silva-138-99-nb-classifier. R (version 3.5.3; R Core Team, 2021), RStudio Team (2020), Tax4Fun2 (Wemheuer et al., 2020) and Kyoto Encyclopedia of Genes and Genomes (KEGG; Kanehisa and Goto, 2000; Kanehisa, 2019; Kanehisa et al., 2023) were used to further analyze the data and predict the functional capabilities of the microbial communities.

## 3 Results and discussion

### 3.1 Batch psychrophilic anaerobic digestion performance

**Figure 1:**
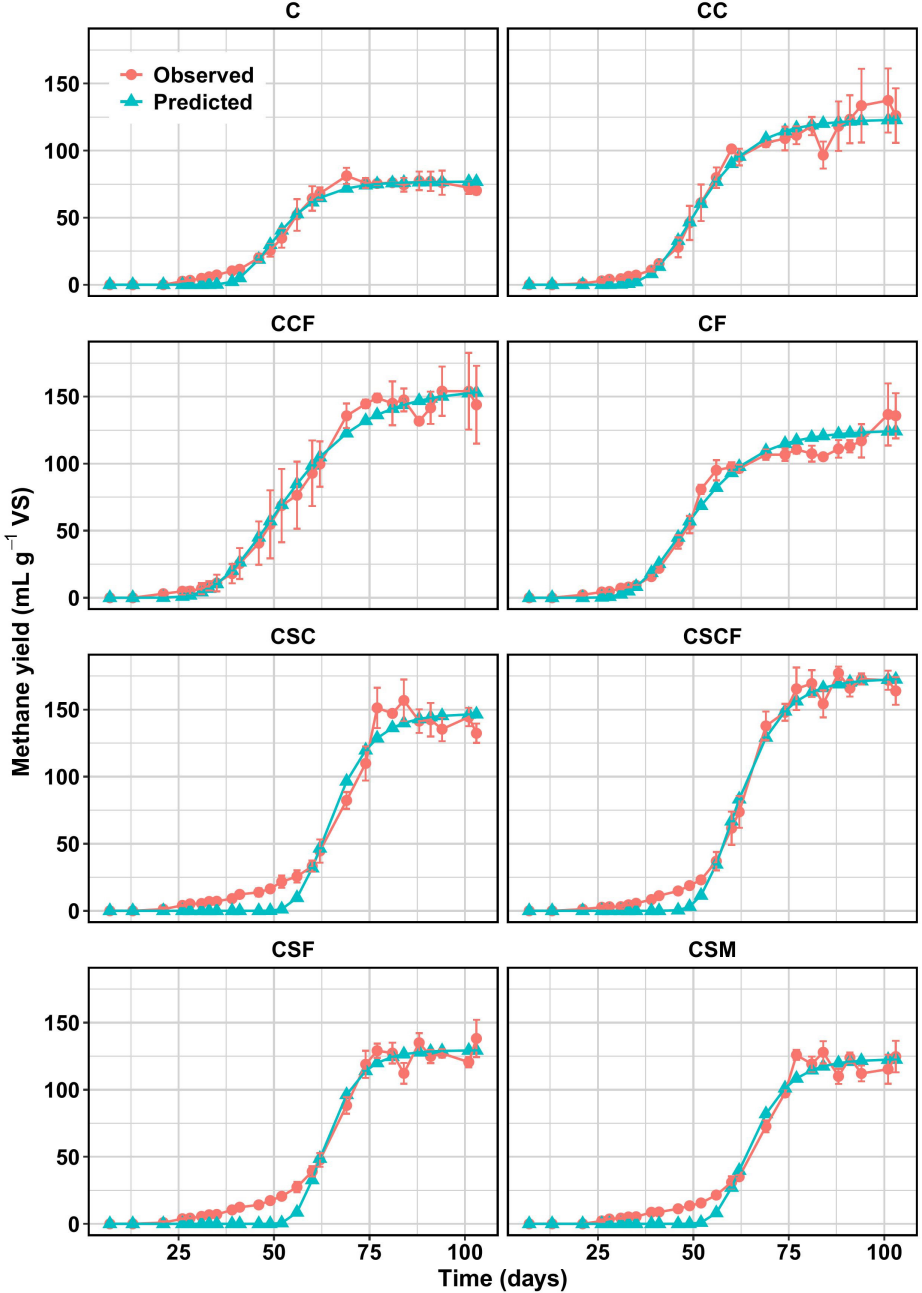
Correlation graphs for observed and predicted methane yields (mL CH_4_ g^−1^ VS; fitted using Modified Gompertz model) from the batch psychrophilic anaerobic digestion cultures (15 °C) treated with various combinations of biological and nano-additives (NPs) over 103 days. C, control; CSM, C + psychrotolerant *S. marcescens* (SM); CSC, CSM + calcium phosphate (CaP) NPs; CSF, CSM + hematite (α-Fe_2_O_3_) NPs; CSCF, CSM + CaP NPs + α-Fe_2_O_3_NPs; CC, C + CaP NPs; CF, C + α-Fe_2_O_3_ NPs; CCF, C + CaP NPs + α-Fe_2_O_3_NPs.

The pH and FOS/TAC ratios obtained for all treatments after the batch culture trials were 6.79–6.91 and 0.4, respectively, which demonstrates that stable PAD occurred with respect to alkalinity and acidity (Nkuna et al., 2021). The inoculum to substrate ratio (ISR) applied in this study was lower than standard recommendations (Holliger et al., 2016). Although this may affect the absolute methane yields, the stable PAD observed suggests that microbial activity was sufficient to support anaerobic conversion. Consequently, the reported methane potentials should be interpreted as indicative values, particularly suitable for relative comparison among treatments. Methane production during the treated and untreated PAD batch culture trials are shown in Figure 1. On average, methane production rapidly increased within the first 70 days of PAD, with the addition of only nanoparticles, compared to the control and SM treated batch cultures. The observed methane production results from the experimental trials that were evaluated using the modified Gompertz model were fitted with the predicted data (*R*^2^ ≥ 0.98), as shown in Table 2. The lag phase results indicated that the nano-additives induced an augmentative effect on methane production by significantly reducing the lag phase compared to the control and SM treated batch cultures. The SM treated batch cultures had significantly longer lag phases compared to the control and treatment with nano-additives alone. Extended lag phase using bioaugmentation with SM may have occurred due to competition with the indigenous AD microbial community or insufficient inoculum size (Akindolire et al., 2022). Nevertheless, higher methane yields were produced by all treated batch cultures after 70 days in comparison to the control. The PAD microorganisms possibly adapted to the new environment and rapidly increased in activity after 70 days (Jákói et al., 2022), as reflected by the exponential increase in methane yield (Figure 1). Furthermore, a reduced lag phase was observed when SM was applied with the combination of both nano-additives compared to the other SM treated batch cultures. Moreover, after 70 days and until the end of the trial, the highest methane yields were produced from digestion with the addition of SM in combination with both nano-additives. These results suggest that the augmentative effect of SM with the nano-additives on methane production possibly occurred only after the establishment of SM during the PAD process.

**Table 2:** Kinetic features of the batch cultures.

**Treatment**	**O (mL CH_4_ g^−1^ VS)**	**P (mL CH_4_ g^−1^ VS)**	**Rm (mL CH_4_ g^−1^ VS d^−1^)**	**λ (d)**	***R* ^2^**
C	70.2 ± 4.9^b^^*^	76.9	3.7	43.0 ± 4.6^a^	0.99
CSM	124.6 ± 20.3^a^	122.9	6.6	55.3 ± 2.5^c^	0.98
CSC	132.4 ± 12.4^a^	147.0	7.8	56.0 ± 2.6^bc^	0.98
CSF	138.2 ± 24.1^a^	129.4	8.1	52.8 ± 1.9^c^	0.98
CSCF	163.9 ± 18.0^a^	173.1	8.3	52.7 ± 4.2^c^	0.99
CC	126.1 ± 35.2^a^	123.3	4.7	39.1 ± 4.7^ab^	0.98
CF	135.7 ± 29.1^a^	125.0	4.1	34.6 ± 4.0^ab^	0.98
CCF	143.9 ± 50.2^a^	156.4	4.1	38.9 ± 7.7^ab^	0.99

O, observed maximum methane yields on day 103; P, predicted maximum methane potential yields on day 103; Rm, simulated maximum rate of methane production; λ, lag phase in days (d); C, control; CSM, C + psychrotolerant *S. marcescens* (SM); CSC, CSM + calcium phosphate (CaP) NPs; CSF, CSM + hematite (α-Fe_2_O_3_) NPs; CSCF, CSM + CaP NPs + α-Fe_2_O_3_NPs; CC, C + CaP NPs; CF, C + α-Fe_2_O_3_ NPs; CCF, C + CaP NPs + α-Fe_2_O_3_NPs.

* Different letter combinations represent significant differences between treatments (*p* < 0.05).

The simulated methane production rates had improved in the nano-additive treated batch cultures and more so with the addition of SM in combination with the nano-additives, compared to the control. Moreover, maximum methane yields from the SM and/or nano-additive treated batch cultures were significantly higher compared to the control. The addition of SM with the respective nano-additives improved maximum methane yields compared to batch cultures treated with nano-additives alone (i.e. CSC>CC; CSF>CF; CSCF>CCF). The biomass of SM added was negligible compared to the total substrate, thus its effect was primarily metabolic. Batch cultures treated with nano-additives alone resulted in higher maximum methane yields compared to treatment with SM alone. Furthermore, the highest maximum methane yields were obtained from CSCF and CCF, rendering a 133.5% and 105.0% increase, compared to the control. These results suggest that the combined stimulating and conductive attributes of the nano-additives possibly contributed to the enhancement of methane production through synergistic interaction with SM and/or the indigenous PAD microbial communities. Zhu et al. (2022) reported a similar augmentative effect of iron oxide (magnetite), calcium peroxide and the combined use of iron oxide and calcium peroxide on mesophilic AD of food waste and sludge, whereby a 5.95, 15.3 and 26.8% increase in methane production was observed, respectively, compared to their control (Zhu et al., 2022).

The maximum methane yield results of the batch cultures in this study were comparable to results from previous batch PAD studies and some mesophilic AD studies conducted under non-augmented conditions (i.e., without the addition of nanoparticles, nutrients, or engineered microbes), however augmented mesophilic AD systems resulted in higher maximum methane yields (Table 3), as expected. Nonetheless, bioaugmentation with SM resulted in a methane yield of 124.6 ± 20.3 mL CH_4_ g^−1^ VS at 15 °C, which was comparable to and higher than previously reported methane yields obtained at 20 °C from bioaugmentation with mesophilic/psychrotolerant consortia (Table 3). The superior performance of SM may be attributed to its ability to produce cold-active enzymes involved in the fermentative stages, thus contributing to the availability of methanogenic precursors. Despite the enhanced maximum methane yields from augmentation with the additives, the lag phase period in this study (35–56 days) was longer compared to other PAD studies that have reported shorter lag phases (0.51–16 days) (Martí-Herrero et al., 2022; Xu et al., 2023). In the present study, the batch bottles were not flushed with nitrogen gas initially and a low ISR was applied, which may have contributed to the overall prolonged lag phase. Moreover, this study utilized a cold-adapted mesophilic inoculum to start-up the batch culture. To reduce the lag phase by improving the affinity between the substrate and inoculum, it is recommended to further investigate the application of a stabilized psychrophilic inoculum (Martí-Herrero et al., 2022) and an ISR between 1 and 4 (Holliger et al., 2016; Koch et al., 2020). Overall, the results indicate that the combined application of the nano-additives, with or without SM, has the potential to enhance methane production at low temperatures (15 °C).

**Table 3:** Comparative assessment of maximum methane yield from various batch psychrophilic and mesophilic anaerobic digestion studies.

**Inoculum**	**Feedstock**	**Treatment**	**Temperature (°C)**	**Methane yield (mL CH_4_ g^−1^ VS)**	**References**
**Psychrophilic**					
Cold-adapted CM and FW inoculum	CM and mixed vegetable FW	Psychrotolerant *S. marcescens*, α-Fe_2_O_3_ NPs and/or CaP NPs	15	70.2 ± 4.9–163.9 ± 18.0^*^	This study
Psychrophilic cattle manure and food waste inoculum	CM and mixed vegetable FW	None	15	74 ± 9	Rama et al. (2024)
Cellulose and natural peptone digestate	Cow manure and corn straw	Cold-tolerant methanogenic consortium seed	20 ± 1	15.40–73.01^*^	Xu et al. (2023)
Mesophilic or psychrophilic cattle manure inoculum	Cattle manure	Mesophilic or psychrophilic cattle manure inoculum	15 ± 1	7.72–14.99^*^	Zhu and Jha (2013)
Corn stalk digestate fed with cellulose and bacterial peptone	Cow manure and corn straw	Mesophilic consortium seed from propionate-degrading reactor	20 ± 1	8–36^*^	Xu et al. (2022)
Psychrophilic pig manure, barley straw and cattle manure digestate	Pig manure, cattle manure and/or barley straw	3,000 m above sea level	15	57.8–152.3	Wei et al. (2014)
Mesophilic cattle manure digestate	Swine manure	Iron oxide and zeolite	15	39.49–126.97^*^	Lu et al. (2018)
Cattle manure anaerobic sludge digestate	Cow manure and/or cheese whey	None	15	140–240	Jaimes-Estévez et al. (2022)
**Mesophilic**					
Mesophilic cattle manure digestate	Swine manure	Iron oxide and zeolite	25	189.20–285.08^*^	Lu et al. (2018)
Mesophilic cattle manure digestate	Swine manure	Iron oxide and zeolite	35	327.14–437.85^*^	Lu et al. (2018)
Anaerobic sewage sludge	Food waste	Calcium carbonate	37 ± 1	120.2	Chen et al. (2015)
Dairy manure digestate	Goat manure	None	36.5	159	Kafle and Chen (2016)
Dairy manure digestate	Horse manure	None	36.5	155	Kafle and Chen (2016)
Cattle dung digestate slurry	Microalgal slurry	Iron oxide NPs (30 mg L^−1^)	37 ± 1	363.34	Rana et al. (2020)
Cattle manure anaerobic sludge digestate	Cow manure and/or cheese whey	None	35	320–600	Jaimes-Estévez et al. (2022)

* First value is the control (untreated).

### 3.2 Microbial community composition

**Figure 2:**
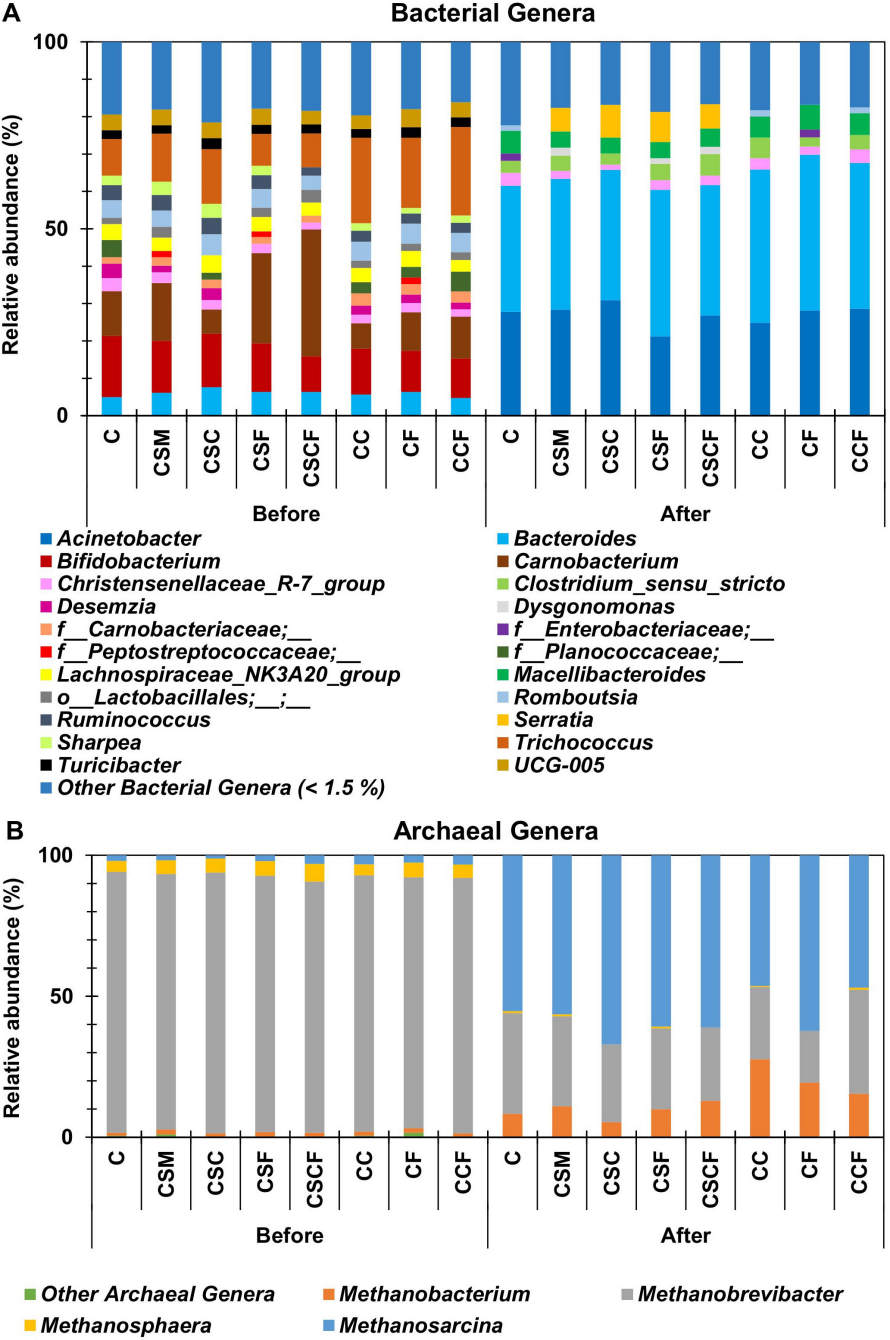
**(A)** Bacterial and **(B)** archaeal taxonomic classification at the genera level before (day 0) and after (day 103) psychrophilic anaerobic digestion batch trials at 15 °C. C, control; CSM, C + psychrotolerant *S. marcescens* (SM); CSC, CSM + calcium phosphate (CaP) NPs; CSF, CSM + hematite (α-Fe_2_O_3_) NPs; CSCF, CSM + CaP NPs + α-Fe_2_O_3_NPs; CC, C + CaP NPs; CF, C + α-Fe_2_O_3_ NPs; CCF, C + CaP NPs + α-Fe_2_O_3_NPs.

The bacterial and archaeal community composition, before and after PAD of CM and FW that was untreated and treated with various combinations of SM and/or nano-additives at 15 °C, is shown in Figure 2. The bacterial and archaeal community composition was similar between all samples, including the control, before PAD (day 0), as expected. However, a major shift occurred in the community composition across all samples after PAD (day 103). This shift resulted from the depletion of oxygen during PAD, which eliminates or diminishes aerobic microorganisms and promotes growth of anaerobic microorganisms that are able to thrive with little to no oxygen (Zakem et al., 2020). The dominant bacterial genera present before PAD were *Carnobacterium* (6.5–33.9%), *Bifidobacterium* (9.5–16.3%), *Trichococcus* (8.5–23.7%) and *Bacteroides* (4.7–7.6%; Figure 2A). These bacteria are typically anaerobic and fermentative (Leisner et al., 2007; Strepis et al., 2020; Kelly et al., 2021), however their diminished presence following PAD suggests that these bacteria were either outcompeted by other dominant bacterial genera or unable to adapt to the low temperatures.

*Acinetobacter* (21.2–30.9%), *Bacteroides* (33.7–41.7%), *Macellibacteroides* (4.2–6.6%), *Clostridium* sensu stricto (2.5–5.8%) and Christensenellaceae_R-7_group (1.4–3.6%) were the dominant bacterial genera after PAD. These bacterial genera play a role in fermentation and acetogenesis (Akindolire et al., 2022; Wei et al., 2022). The bacterial genera that were present in all the samples before and after PAD were *Bacteroides* and Christensenellaceae_R-7_group. This demonstrates their ability to persist and adapt to changing environments (i.e., in terms of low temperature, oxygen depletion as well as in the presence of SM and/or nano-additives). Besides *Acinetobacter*, the observed dominant and persistent bacterial genera can produce various volatile/short chain fatty acids such as acetate, butyrate, propionate, succinate and/or lactate through fermentation and acetogenesis (Nishiyama et al., 2009; Ueki et al., 2011; Jabari et al., 2012; Wei et al., 2022). On the other hand, *Acinetobacter* possibly played a role in fermentation and acetogenesis by utilizing acetate and various other carbon sources for growth (Kim et al., 1997). Furthermore, *Bacteroides* and *Acinetobacter* are electroactive bacteria that are capable of enhancing methane production via DIET (Harirchi et al., 2022; Zheng et al., 2023). Other PAD studies have also reported the increased relative abundance of *Bacteroides, Clostridium* and *Acinetobacter*, which suggests that these bacteria play an important role in methane production under psychrophilic conditions (Xu et al., 2023; Yan et al., 2023; Zheng et al., 2023).

The archaeal community was primarily composed of *Methanobrevibacter* (89.0-92.5%), *Methanosphaera* (3.9–6.2%) and *Methanosarcina* (1.2–3.2%) before PAD across all samples, however by the end of the trial all samples were dominated by *Methanosarcina* (46.4–67.0%), thereafter *Methanobrevibacter* (18.3–36.9%) and *Methanobacterium* (5.3–27.6%; Figure 2B). *Methanobrevibacter* and *Methanobacterium* utilize hydrogen (H_2_) and carbon dioxide (CO_2_) as electron acceptors during hydrogenotrophic methanogenesis, while electrotrophic *Methanosarcina* can utilize H_2_/CO_2_, acetate, methanol and/or methylamines during hydrogenotrophic, acetoclastic and methylotrophic methanogenesis, respectively (Akindolire et al., 2022). Previous reports have indicated that the acetoclastic pathway for methanogenesis is more thermodynamically favorable under psychrophilic conditions (Tiwari et al., 2021; Akindolire et al., 2022; Yan et al., 2023). Hence, the increased presence of *Methanosarcina* possibly stimulated the acetoclastic methanogenesis pathway under psychrophilic conditions.

A visible difference between the samples after PAD, based on the taxonomic classification, includes the dominant presence of *Serratia* in the samples that were initially inoculated with SM. These results indicate that by the end of the PAD trial, SM had established itself within the PAD microbial community without being outcompeted by other microorganisms. A negligible difference (0.18%) was observed between CSM and CSCF after the PAD trial in terms of the relative abundance of *Serratia*. The results suggest that both nano-additives improved the efficiency of SM and possibly other microorganisms despite the relative abundance of SM during PAD. Other studies that were conducted under mesophilic and thermophilic temperatures have reported similar findings, whereby the combination of bioaugmentation and nano-additives enhanced methane production compared to bioaugmentation alone (Xiao et al., 2019; Laikova et al., 2023). These results further support the proposition that the establishment of SM within the PAD microbiome was crucial for its augmentative effect with the nano-additives to occur on methane production. Moreover, significant positive correlations were observed between *Serratia, Acinetobacter, Bacteroides, Clostridium, Dysgonomonas, Methanobacterium, Methanosarcina* and the maximum methane potentials from this study (Supplementary Figure 3). This key finding suggests that syntrophic relationships possibly existed between these diverse microbial groups, thus contributing to methane production.

### 3.3 Microbial community diversity

**Figure 3:**
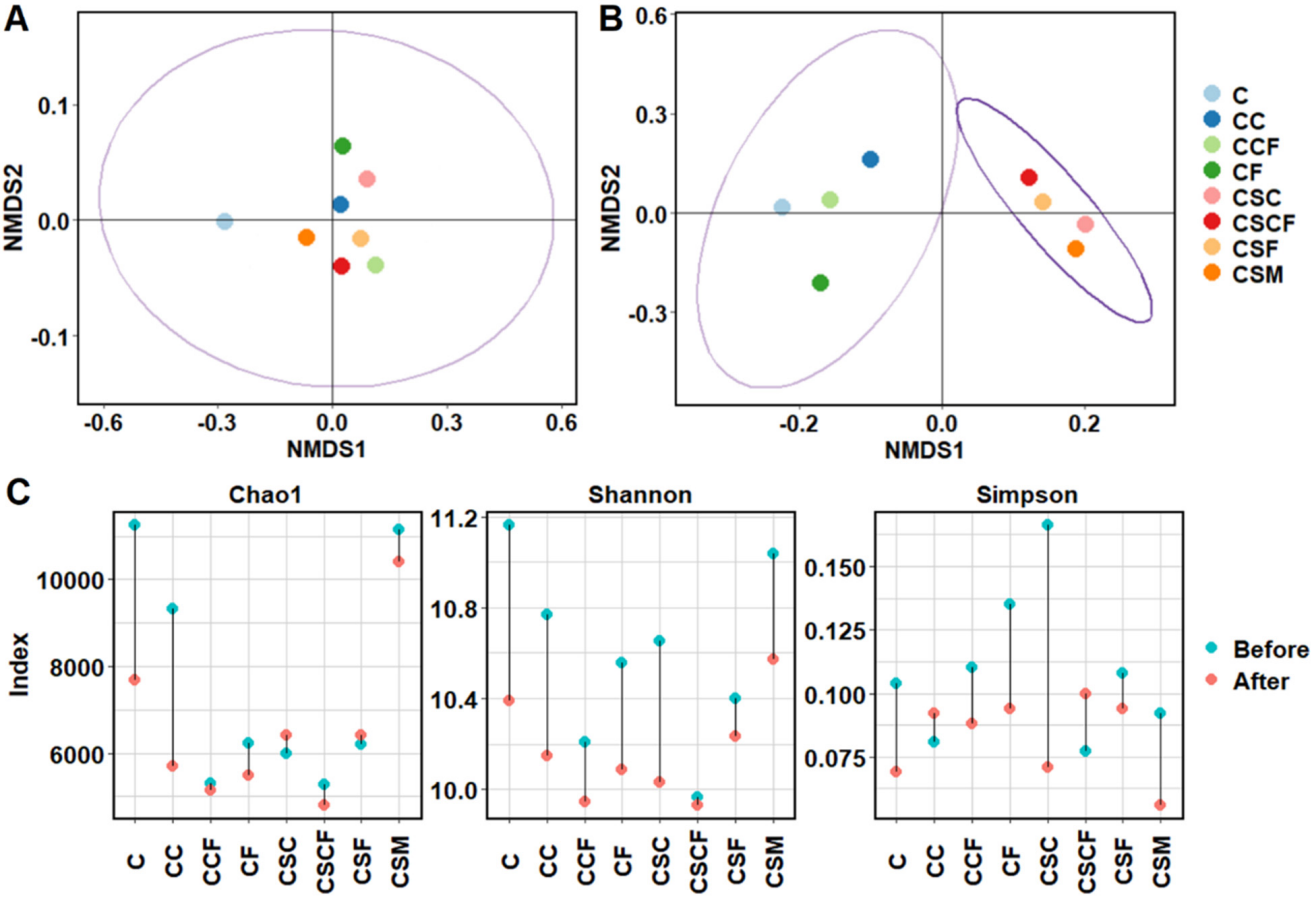
NMDS plot of the microbial communities in the treated and untreated batch cultures before (day 0) **(A)** and after (day 103) **(B)** psychrophilic anaerobic digestion (PAD) based on Bray Curtis metrics (ellipses were plotted at 95% confidence). Alpha diversity indices (Chao1, Shannon and Simpson) of the treated and untreated batch cultures **(C)**. C, control; CSM, C + psychrotolerant *S. marcescens* (SM); CSC, CSM + calcium phosphate (CaP) NPs; CSF, CSM + hematite (α-Fe_2_O_3_) NPs; CSCF, CSM + CaP NPs + α-Fe_2_O_3_NPs; CC, C + CaP NPs; CF, C + α-Fe_2_O_3_ NPs; CCF, C + CaP NPs + α-Fe_2_O_3_NPs.

A distinct change in microbial community diversity was observed before and after PAD. This can be attributed to the transition from aerobic to anaerobic conditions (Rama et al., 2024). The NMDS plot of the microbial community beta diversity, based on Bray-Curtis metrics, indicates that the microbial communities before PAD clustered together, as expected (Figure 3A). Interestingly, after PAD, the microbial communities remained clustered together following treatment with the nano-additives alone (Figure 3B), while treatment with SM and nano-additives significantly altered the core diversity of the microbial communities, despite the augmentative effect of the treatments on methane production. Low doses of calcium (20 mg L^−1^) were found to enhance methane production from digestion of long-chain fatty acids with little impact on microbial community diversity compared to high doses (2,500 mg L^−1^; Liu et al., 2021). A previous study reported that calcium phosphate can influence the microbiome and promote production of VFAs by members of the Firmicutes and *Bacteroides* bacterial taxa (Fuhren et al., 2021). The findings of those studies support the augmentative effect of the CaP NPs during PAD observed in this study and its minimal impact on diversity. Other contrasting studies have reported a shift in microbial community from augmentation of mesophilic anaerobic digesters with α-Fe_2_O_3_ NPs and/or calcium peroxide nanoparticles as well as bioaugmentation with microbial consortia (Wang et al., 2016; Xu et al., 2022; Zhu et al., 2022). A possible syntrophic relationship may have been established via DIET between some of the fermentative/acetogenic bacteria and methanogens with the aid of the α-Fe_2_O_3_ NPs. Previous studies have reported on similar findings whereby α-Fe_2_O_3_ NPs promoted methane production via DIET between syntrophic microorganisms at mesophilic temperatures (Wang et al., 2016; Aguilar-Moreno et al., 2020; Rana et al., 2020; Zhu et al., 2022). As such, the CaP NPs and α-Fe_2_O_3_ NPs may have provided a stimulating benefit to the microorganisms during enhanced PAD with minimal effect on the microbial community compared to the control. In contrast, the addition of SM (with or without the nano-additives) resulted in its establishment in the microbial community, thus altering the community structure and diversity. Nonetheless, enhanced methane production was observed despite the altered microbial diversity due to the presence of SM.

The alpha diversity indices of the microbial communities before and after batch PAD are shown in Figure 3C. The species richness (Chao1), diversity (Shannon) and evenness (Simpson) of the microbial communities mostly decreased after PAD, most likely due to the decrease in temperature and anaerobic conditions, which provides a niche environment for certain microorganisms (Zakem et al., 2020). Although treatment with SM and CaP NPs or α-Fe_2_O_3_ NPs enhanced species richness after PAD in the microbial communities of CSC and CSF, treatment with all three additives (CSCF), which gave the highest methane production potential, resulted in the lowest microbial diversity after PAD. These results suggest that a stable and co-operative microbial community with lower diversity was prevalent during enhanced methane production with the combined treatment.

### 3.4 Predicted functions of the microbial communities

**Figure 4:**
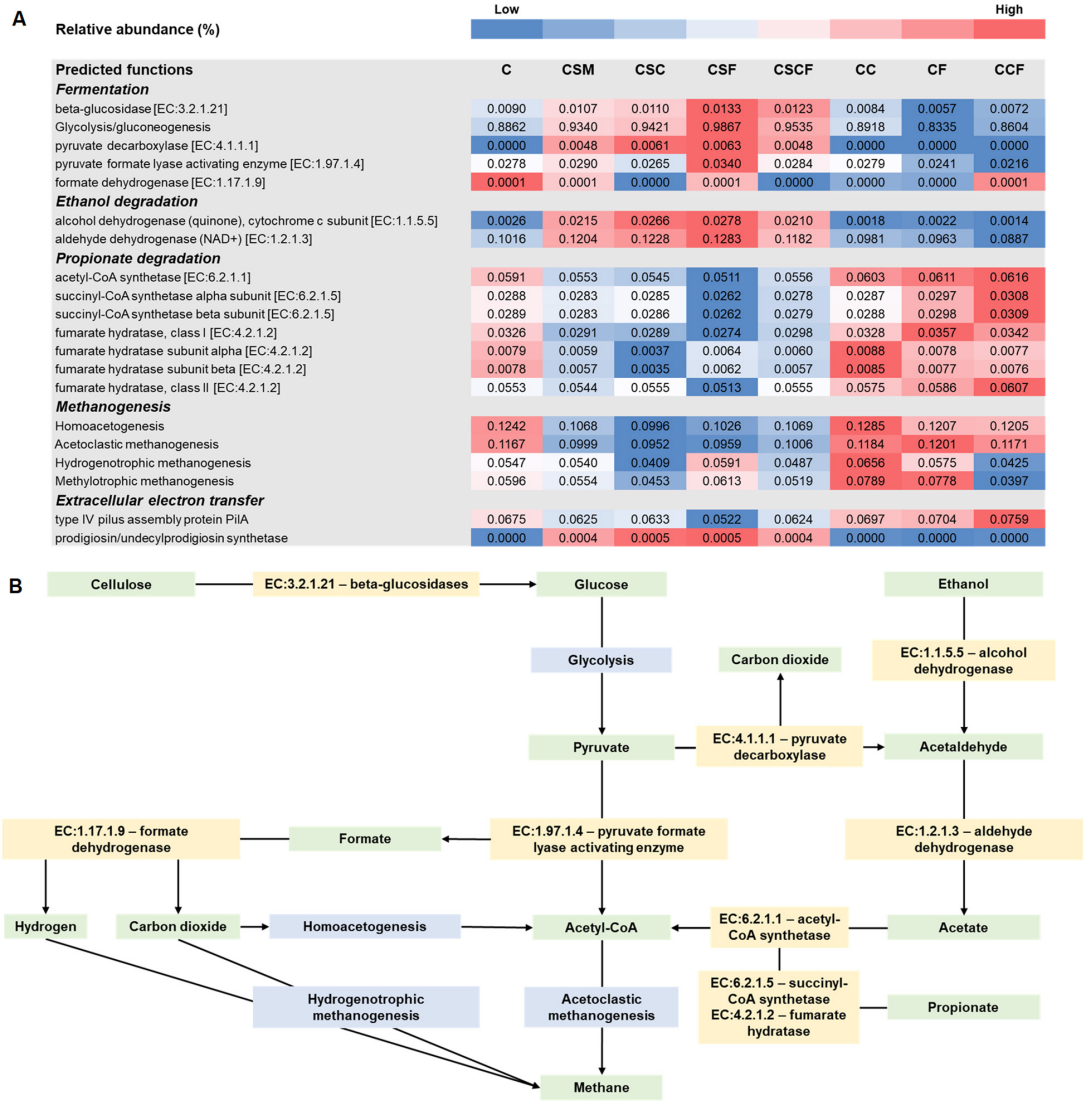
**(A)** Predicted functional capabilities of the batch culture microbial communities after psychrophilic anaerobic digestion (day 103). **(B)** Diagram of predicted functions in metabolic pathways. C, control; CSM, C + psychrotolerant *S. marcescens* (SM); CSC, CSM + calcium phosphate (CaP) NPs; CSF, CSM + hematite (α-Fe_2_O_3_) NPs; CSCF, CSM + CaP NPs + α-Fe_2_O_3_NPs; CC, C + CaP NPs; CF, C + α-Fe_2_O_3_ NPs; CCF, C + CaP NPs + α-Fe_2_O_3_NPs.

In SM treated batch cultures, the relative gene abundance for predicted functions in fermentation were enriched compared to the other treatments (Figure 4). In another study, SM displayed hydrolytic and acidogenic capabilities during mesophilic AD (Obi et al., 2020). This suggests that SM likely played an important role in the initial stages of PAD. Moreover, high relative gene abundances for pyruvate decarboxylase (EC:4.1.1.1), alcohol dehydrogenase (EC:1.1.5.5) and aldehyde dehydrogenase (EC:1.2.1.3) were detected in SM treated batch cultures. These enzymes are responsible for the conversion of pyruvate and ethanol to acetaldehyde, and acetaldehyde to acetate, respectively (Liu et al., 2025). Furthermore, batch cultures treated with the individual and combined nano-additives alone had enriched predicted functional capabilities associated with propionate degradation. The results suggest that the additives may have contributed to the acetogenesis stage, hence it is recommended that future work validate this potential role of the additives through co-culture studies with cellulose, propionate or ethanol as substrates. The enhanced methane potentials from the treated batch cultures during methane production could possibly be attributed to its role in alleviating the rate-limiting stages, which are often the hydrolysis stages in mesophilic AD and VFA accumulation stages of PAD (Obi et al., 2020; Zheng et al., 2023).

Through the fermentation and acetogenesis stages, organic substrates are converted to acetate and acetyl-CoA (Zheng et al., 2023). All microbial communities from the treated and untreated batch cultures had higher relative abundance of genes associated with the homoacetogenesis and acetoclastic methanogenesis pathways compared to the hydrogenotrophic and methylotrophic pathways. These results support the proposition that acetoclastic methanogenesis was the dominant pathway leading to methane production during PAD. However, the SM treated batch cultures had relatively lower predicted functional capabilities in methanogenesis despite their higher methane yields compared to the batch cultures treated with nano-additives alone. Some strains of *S. marcescens* are capable of producing a red pigment called prodigiosin, which was previously found to form conductive films that enhanced electron flow in bioelectrical systems (Zani et al., 2024). In this study, only microbial communities from SM treated batch cultures had a high relative gene abundance for prodigiosin synthetase. Growth of SM on an NA plate confirmed that it was a pigmented strain (Supplementary Figure 1). These results suggest that SM and prodigiosin possibly played a role in stimulating EET during PAD, which may explain the higher methane yields observed from the SM treated batch cultures. However, further investigation of the individual effect of prodigiosin on methane yields is recommended to provide insight into its role in PAD.

In a previous study, coal slime containing conductive materials increased type IV pilus protein PilA gene abundance, and enhanced methane yields from mesophilic AD of chicken manure (Zhao et al., 2024). Besides their roles in microbial adhesion, biofilm formation and motility, the type IV pilus assembly protein PilA and its associated monomers can form electrically conductive pili (e-pili) that play an important role in long-distance EET (Lovley and Holmes, 2020). In this study, treatment with the nano-additives alone, especially in combination, predominantly increased the relative gene abundance of type IV pilus assembly proteins compared to the control and SM treated batch culture communities. Moreover, the nano-additives used in this study had elevated conductivity (Supplementary Table 1) compared to previously reported conductivity of e-pili (2-20 μS cm^−1^; Kumar et al., 2021). These results suggest that the nano-additives augmented methane yields possibly due to DIET interactions between syntrophic, electron-donating bacteria (*Acinetobacter, Clostridium* and *Sedimentibacter*) and electron-accepting methanogens (*Methanosarcina*) by acting as electron shuttles (Wang et al., 2023). Taken together, these findings support the superior performance observed from the augmented batch cultures, which were possibly linked to enhancement of key AD functions and DIET interactions. However, it should be noted that predicted functional analysis provides indirect evidence for DIET (Khalid et al., 2025). Therefore, co-culture and mixed microbial community studies incorporating methods such as metatranscriptomics, metaproteomics, isotope tracing, electrochemical techniques or cellular characterization via electron microscopy are recommended to validate the direct role of the additives in DIET during PAD.

## 4 Conclusions

The combination of calcium phosphate and hematite nano-additives, and psychrotolerant *S. marcescens* augmented methane production via psychrophilic anaerobic digestion of cattle manure and food waste at 15 °C. This was attributed to the biostimulating and conductive properties of the nano-additives on various psychrophilic anaerobic digestion metabolic pathways. Furthermore, the results of this study showed that psychrotolerant *S. marcescens* was a prospective candidate for bioaugmentation due to its diverse metabolic capabilities, particularly during hydrolysis. It was also found that the additives enriched the genetic potential of the resident microbial communities in relation to the anaerobic digestion metabolic pathways and extracellular electron transfer, which may have contributed to the enhanced methane yields during augmented psychrophilic anaerobic digestion. While bioaugmentation with psychrotolerant *S. marcescens* did not improve methane production until it had established itself within the microbial community, its combined application with the nano-additives significantly enhanced its augmentative effect on methane production by the end of the trial. However, treatment with combined nano-additives alone resulted in a reduced lag phase and high maximum methane potential yields. The findings from this study provide a basis for further research and optimization of methane production via psychrophilic anaerobic digestion using psychrotolerant *S. marcescens* and/or the nano-additives.

## Data Availability

The raw sequence data is available in the National Center for Biotechnology Information (NCBI) GenBank under Bio Project ID PRJNA996176 (https://www.ncbi.nlm.nih.gov/sra/PRJNA996176). Further inquiries can be directed to the corresponding author.
