# Supplementary material for: Augmentation of psychrophilic anaerobic digestion with psychrotolerant Serratia marcescens, calcium phosphate (CaHPO4·2H2O) and hematite (α-Fe2O3) nano-additives
Source: Front Microbiol. 2026 Feb 25;17:1756298. doi: 10.3389/fmicb.2026.1756298 (PMC12975899; doi:10.3389/fmicb.2026.1756298)
Supplement: Supplementary file 1 [file Data_Sheet_1.pdf]

Supplementary Material

Augmentation of psychrophilic anaerobic digestion with psychrotolerant *Serratia marcescens*, calcium phosphate ($CaHPO_4{\cdot}2H_2O$) and hematite (α-Fe_2O_3) nano-additives

Haripriya Rama[1,2*], Busiswa Ndaba[3], Mokhotjwa Simon Dhlamini[2], Malik Maaza[4,5], Nicolene Cochrane[6], Ashira Roopnarain[1,7]

[1] Microbiology and Environmental Biotechnology Research Group, Agricultural Research Council – Natural Resources and Engineering, Pretoria, South Africa

[2] Department of Physics, College of Science, Engineering and Technology, University of South Africa – Florida Campus, Johannesburg, South Africa

[3] Institute for Catalysis and Energy Solutions, College of Science, Engineering and Technology, University of South Africa – Florida Campus, Johannesburg, South Africa

[4] Nanosciences African Network Materials Research Department, iThemba LABS, National Research Foundation of South Africa, Cape Town, South Africa

[5] UNESCO-UNISA iTLABS/NRF Africa Chair in Nanosciences-Nanotechnology, College of Graduate Studies, University of South Africa, Pretoria, South Africa

[6] AGRIMETRICS, Agricultural Research Council – Biometry, Pretoria, South Africa

[7] Department of Environmental Sciences, College of Agriculture and Environmental Sciences, University of South Africa – Florida Campus, Johannesburg, South Africa

*** Correspondence:**
Haripriya Rama
ramah@arc.agric.za

Supplementary Material

Supplementary Table S1. Summarized characteristics of the nanoparticles used in this study (Rama et al., 2025).

**Nanoparticle**	**Particle size (nm)**	**Crystallite size (nm)**	**Surface area (m^2 g^{-1})**	**Pore diameter (nm)**	**Zeta potential (mV)**	**Conductivity at 25 °C (μS cm^{-1})**	**Elemental composition**
Calcium phosphate	30.9 ± 9.8	70.65	47.95 ± 0.09	12.36	–16.3 ± 4.87	148	Ca, P, O, C
Hematite	16.5 ± 6.6	35.04	22.16 ± 0.02	11.16	–24.0 ± 6.23	55	Fe, O, C

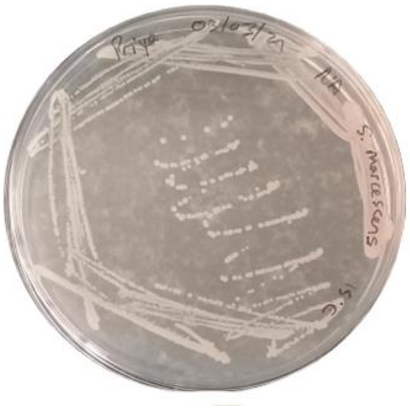

Supplementary Figure S1. Growth of psychrotolerant *Serratia marcescens* at 15 °C on nutrient agar.

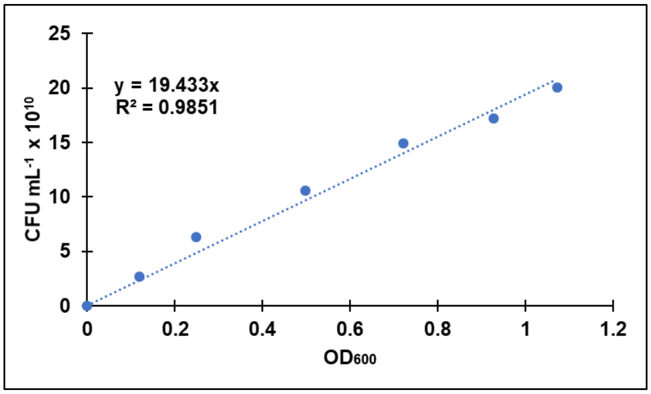

Supplementary Figure S2. Standard concentration regression line for psychrotolerant *Serratia marcescens*.

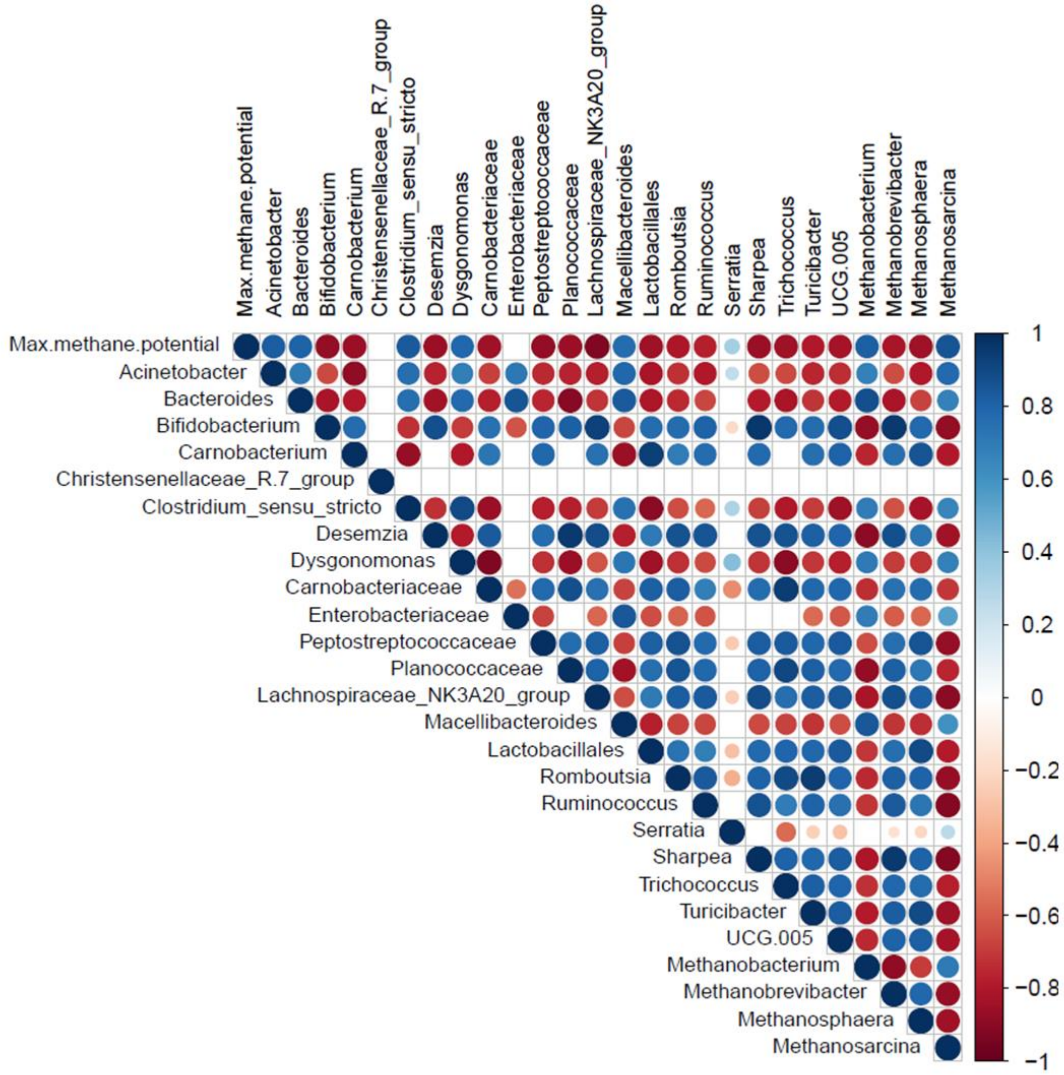

Supplementary Figure S3. Spearman's rank correlogram of significant correlations ($p<0.05$) between dominant bacterial and archaeal taxa, and maximum methane potential across treatments. Larger circles indicate stronger correlations, while smaller circles indicate weaker correlations.

References

Rama, H., Ndaba, B., Azizi, S., Vatsha, B., Dhlamini, M. S., Maaza, M., et al. (2025). Sclerocarya birrea-mediated calcium phosphate and iron oxide nanoparticles: Effect of extraction time and annealing temperature on physical properties. *Heliyon* 11, e44021. doi: 10.1016/j.heliyon.2025.e44021
